# [Retracted] Upregulated microRNA-671-3p promotes tumor progression by suppressing forkhead box P2 expression in non-small-cell lung cancer

**DOI:** 10.3892/mmr.2026.13829

**Published:** 2026-02-16

**Authors:** Zhi-Ying Li, Zi-Zhou Zhang, Hui Bi, Qiu-Di Zhang, Su-Juan Zhang, Lin Zhou, Xiao-Qin Zhu, Jun Zhou

Mol Med Rep 20: 3149–3159, 2019; DOI: 10.3892/mmr.2019.10563

Following the publication of this paper, it was drawn to the Editor's attention by a concerned reader that, in addition to the duplication of a pair of data panels in Fig. 7A, flow cytometric (FCM) assay data featured in Figs. 2D and 6B were strikingly similar to FCM data which were ultimately published in a number of other papers in different journals that were written by different authors at different research institutes, including a paper that was submitted on an earlier date to the journal *Experimental and Therapeutic Medicine.*

Owing to the fact that the contentious data in the above article were found to be strikingly similar to data that have appeared elsewhere in other papers in the scientific literature, the Editor of *Molecular Medicine Reports* has decided that this paper should be retracted from the Journal. The authors were asked for an explanation to account for these concerns, but the Editorial Office did not receive a reply. The Editor apologizes to the readership for any inconvenience caused.

